# Energy dissipation efficiency as a new variable in the empirical correlation of total dissolved gas

**DOI:** 10.1038/s41598-021-86144-y

**Published:** 2021-04-01

**Authors:** Jingying Lu, Xiaolong Cheng, Zhenhua Wang, Ran Li, Jingjie Feng, Kefeng Li, Zhongluan Yan

**Affiliations:** 1 Sichuan University State Key Laboratory of Hydraulics and Mountain River Engineering, Chengdu, Sichuan China; 2 China Three Gorges Corporation, Beijing, China; 3 China Three Gorges Projects Development Co., Ltd, Chengdu, China

**Keywords:** Environmental impact, Hydrology

## Abstract

Total dissolved gas (TDG) supersaturation, which occurs during dam spilling, may result in fish bubble disease and mortality. Many studies have been conducted to identify the factors pertaining to TDG generation, such as the spilling discharge and tailwater depth. Additionally, the energy dissipation efficiency should be considered due to its effect on the air entrainment, which influences the TDG generation process. According to the TDG field observations of 49 cases at Dagangshan and Xiluodu hydropower stations, the TDG was positively related to the energy dissipation efficiency, tailwater depth and discharge per unit width. A correlation between the generated TDG level and these factors was established. The empirical equations proposed by the USACE were calibrated, and the TDG level estimation performance was compared with the established correlation for 25 spillage cases at seven other dams. Among the considered cases, the standard error of the TDG estimation considering the energy dissipation efficiency was 5.7%, and those for the correlations obtained using the USACE equations were 13.0% and 10.0%. The findings indicated that the energy dissipation efficiency considerably influenced the TDG level, and its consideration helped enhance the precision of the TDG estimation. Finally, the generality of this approach and future work were discussed.

## Introduction

Supersaturated total dissolved gas (TDG) has gradually gained public attention due to its adverse effect on aquatic biota. Supersaturated TDG can lead to bubble disease and mortality of fish^1–6^ and cause considerable damage to the eco-environment. In general, supersaturated TDG levels can be attributed to dam spillage^7^, waterfalls and similar phenomena. TDG supersaturation is always observed in rivers^8,9^. Many studies have been conducted to mitigate the harmful effects of TDG supersaturation. Accurate predictions of TDG saturation associated with dam spilling are the basis for further research on TDG mitigation measures. TDG generation from spillage is the combination of an air–water transfer process and dam spilling process. Specifically, dam spilling results in a large amount of air being entrained in a stilling basin, and in this high-pressure situation, air starts to dissolve, which produces supersaturated TDG flows.

The methods to predict the supersaturated TDG generation can be divided into 3 types: mechanical models, numerical models and empirical equations. Research on the TDG mechanical models is generally based on the gas transfer process and physical spilling process. However, owing to insufficient knowledge of these processes, certain parameters may be neglected in the TDG prediction, leading to an increase in the error of the TDG prediction and restriction of the application range of the model^10–18^. Moreover, considerable calculations must be performed when implementing numerical models, and limited understanding of the two-phase flow movement, bubble transfer process and bubble density distribution may reduce the generality of such models^19–23^. Recently, alternative methods such as neural networks and high-order response surface methods have been used to model the generation of TDG by spilling^24,25^.

Research on empirical equations is scarce, although such equations provide a convenient and rapid way to predict the TDG levels by simply combining certain spilling factors. The Columbia Basin Research School of Fisheries fitted the TDG equation with hyperbolic and exponential relationships considering the spill flow, supersaturation conditions and parameters calibrated based on information from seven different projects. Nevertheless, this TDG correlation considered only the spilling flow, with the standard error ranging from 1.5% at McNary to 8.5% at the Dalles dam^26^. Later, two empirical TDG correlations based on specific flow discharge and tailwater depth values were formulated by the US Army Corps of Engineering according to the TDG measurements collected from the Columbia and Snake River projects^27^. In the established empirical equations, the change in the TDG level $\left( {\Delta G = G_{s} - G_{eq} } \right)$ was a function of the specific discharge $\left( {q_{s} } \right)$ and tailwater depth $\left( {h_{t} } \right)$, as follows:

1 $$ \Delta G = c_{1} h_{t} \left( {1 - e^{{ - c_{2} q_{s} }} } \right) + c_{3} $$

2 $$ \Delta G = c{}_{1}h_{t}^{{c_{2} }} q_{s}^{{c_{3} }} + c_{4} $$

where $G_{s}$ is the TDG level associated with dam spilling (%); $G_{eq}$ is the TDG saturation at the local barometric pressure (with the saturation maintained at 100%); $c{}_{1}$, $c{}_{2}$, $c{}_{3}$ and $c{}_{4}$ are undetermined coefficients. The specific discharge ($q_{s}$) was determined from the flow-weighted discharge, as indicated in Eq. (3)

3 $$ q_{s} { = }\frac{{\sum\limits_{i = 1}^{nb} {Q_{i}^{2} } }}{{\sum\limits_{i = 1}^{nb} {Q_{i}^{{}} } }} $$

where $Q_{i}^{{}}$ denotes the discharge of the flood release structure $i$ ($nb$ is the number of flood release structures).

However, several factors were not extensively considered, and the coefficients were recalibrated by field observations. These issues limited the development of the empirical equations. In this study, the relations between the generated TDG level and the energy dissipation efficiency, discharge per unit width and tailwater depth were explored based on TDG measurements in the Dagangshan and Xiluodu projects. The correlation between the TDG level and energy dissipation efficiency was established. Furthermore, the empirical equations proposed by the USACE were calibrated using the measurements of Dagangshan and Xiluodu. A comparison of the TDG estimation performance with and without the energy dissipation efficiency was conducted based on data from seven projects to demonstrate the significance of the energy dissipation efficiency.

## Results and discussion

### TDG measurements in Dagangshan and Xiluodu

The TDG observations from the Dagangshan and Xiduodu dams are shown in Table 1. Due to upper dam spilling, all the forebay TDG levels are higher than 100%, especially at the Dagangshan hydropower station. The TDG levels downstream of Xiluodu and Dagangshan are 113–125% and 117–141%, respectively.

**Table 1:** TDG observations for the Dagangshan and Xiduodu dams.

Case	Project	Distance between dam and observation location (km)	Release structure	Spilling rate, $Q_{s}$ (m^3^/s)	Power flow, $Q_{P}$ (m^3^/s)	Forebay water elevation (m)	Dam downstream water elevation (m)	Forebay TDG, $G_{f}$ (%)	Observed TDG, $G$(%)
1–8	Dagangshan	1.0	Discharge tunnel	713–2420	269–1350	1124–1129	954–960	108–115	117–124
9–15			Bottom orifice	2584–2680	524–1330	1121–1125	959–960	110–113	132–138
16–19			Bottom orifice and discharge tunnel	1690–2663	775–1230	1122–1123	958–959	113	122–141
20	Xiluodu	4.2	Four bottom orifices	5414	7503	5786	387	125	104
21			Triple bottom orifices	4083	7463	578	385	123	104
22–40			Single bottom orifice	1463–1538	3262–6760	591–600	381–384	113–118	104–107
41–49			Double bottom orifices	3005–3039	2145–6626	595–597	382–385	121–123	106–109

### Variable selection to express the relationship with TDG

As mentioned, the spilling discharge, specific discharge and tailwater depth are the main factors generally used to predict the generated TDG level. The specific discharge and spilling discharge increase the hydrodynamic pressure in the stilling basin and reflect the retention time of the flow in a high-pressure stilling basin. Moreover, the tailwater depth can represent the hydrostatic pressure. Therefore, the specific discharge and tailwater depth are positively related to the generated TDG saturation.

However, the selection of these factors involves certain limitations. First, the spilling discharge and specific discharge are only applicable for dams with similar flood release structures, and these parameters cannot reflect the effect of the engineering characteristics in different flood release structures on the TDG level. In engineering practice, flood discharge structures of different types (e.g., surface orifices, middle orifices, and discharge tunnels) or the same type but with various engineering characteristics are commonly found in one dam. Second, the forebay TDG level is generally considered to be one of the initial conditions associated with the TDG variation process. Therefore, the forebay TDG level should replace the TDG level at atmosphere pressure. Furthermore, no parameter that represents the air entrainment or air–water transfer is considered. In this case, the energy dissipation efficiency should be selected due to its positive relation with the air entrainment^28^.

According to this rationale, the tailwater depth ($h_{t}$), discharge per unit width ($q$), energy dissipation efficiency ($E$) and forebay TDG level ($G_{f}$) were selected to estimate the TDG level:

4 $$ \frac{{G_{s} - G_{f} }}{{G_{f} }} = f_{1} (h_{t} ,E,q) $$

where $G_{s}$ is the TDG level associated with spilling (%). The discharge per unit width ($q$) can be defined as in Eq. (5).

5 $$ q = \frac{{Q_{s} }}{{B_{so} }} $$

where *Q*_*s*_ is the spilling flowrate (m^3^/s); $B_{so}$ is the width of the flood discharge structure (m).

**Figure 1:**
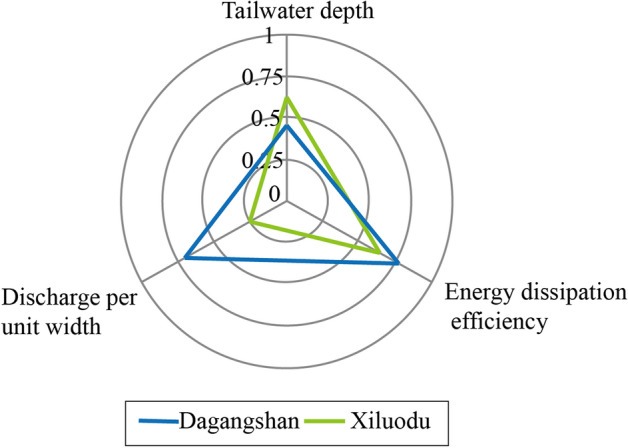
Correlation coefficient between TDG level and selected variables.

TDG measurements in the Dagangshan and Xiluodu projects (Table 1) were used to examine the correlation coefficients between the generated TDG and selected variables: tailwater depth ($h_{t}$), discharge per unit width ($q$), and energy dissipation efficiency ($E$). The result is presented in Fig. 1. The energy dissipation, the discharge per unit and tailwater depth are all relevant to the TDG production in Dagangshan and the energy dissipation efficiency is significant in the TDG results with a correlation coefficient of 0.76. However, in the Xiluodu project, the effect of the energy dissipation efficiency and tailwater depth are larger than the discharge per unit in TDG generation. The factors exhibit different relevance degrees with the TDG in the different projects. Previous studies on the TDG correlation mainly focused on the release structure such as the spillway, owing to which, several factors could not be extensively considered. To establish a more reasonable TDG estimation method, the energy dissipation efficiency is adopted in this study.

### TDG relationship with the energy dissipation efficiency

**Figure 2:**
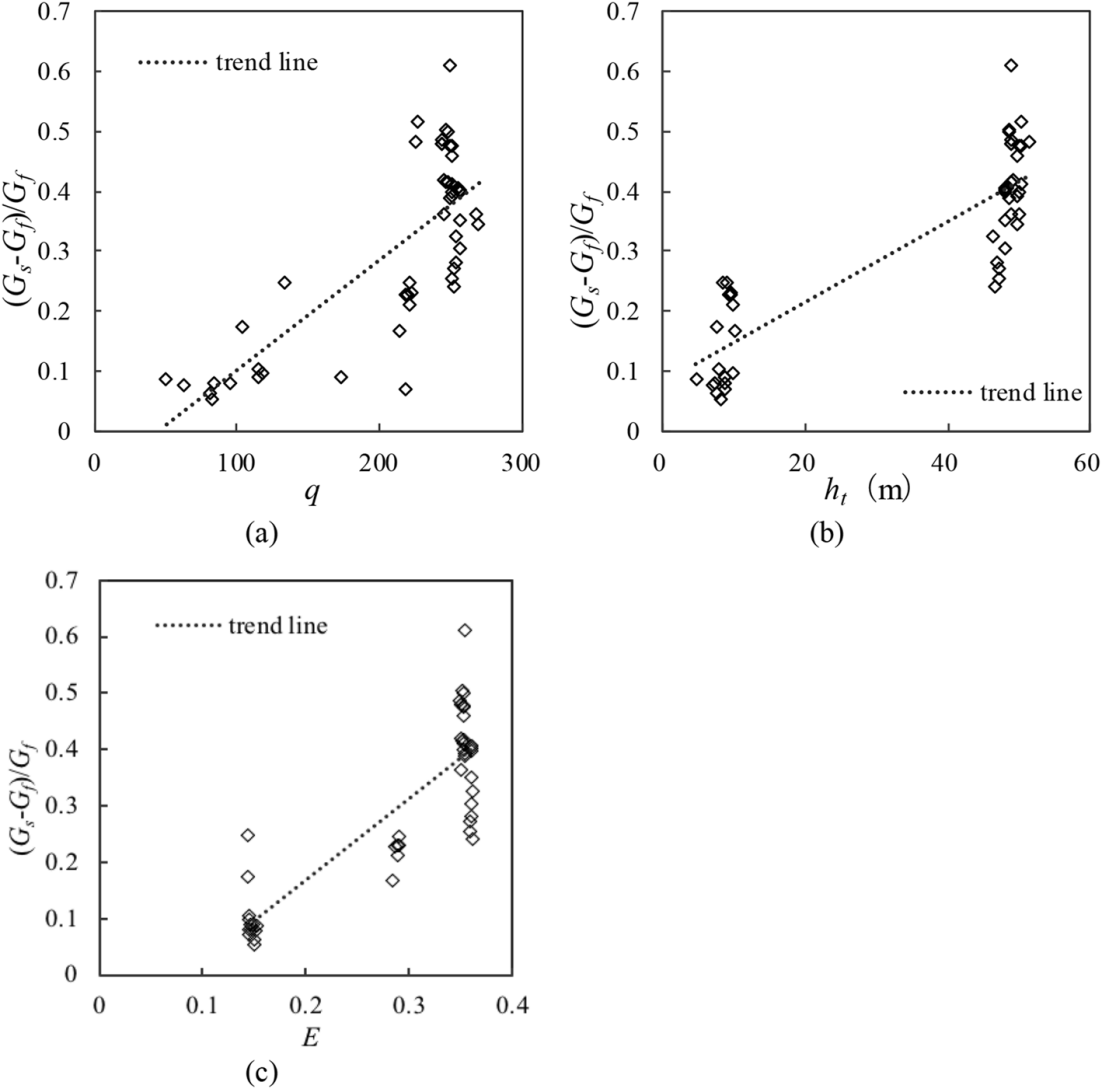
Relationship between $(G_{s} - G_{f} )/G_{f}$ and parameters in Eq. (6): (**a**) tailwater depth ($h_{t}$); (**b**) discharge per unit width ($q$); (**c**) energy dissipation efficiency ($E$).

The relationships between the variation in the TDG $\left( {(G_{s} - G_{f} )/G_{f} } \right)$ and the parameters in Eq. (4) are shown in Fig. 2. The discharge per unit width ($q$) and tailwater depth ($h_{t}$) affect the TDG generation. The variation in the TDG increases with the discharge per unit width ($q$) and tailwater depth ($h_{t}$) (Figs. 2a, b), and this result is consistent with those of the existing studies^3,29^. Moreover, the change in the TDG level is positively correlated with the energy dissipation efficiency ($E$). In particular, in stilling basins, the energy dissipation mainly occurs in an energy cascade process from macroscale to microscale eddies. An intense macroscale eddy leads to a high energy dissipation efficiency and considerable air entrainment^28^. The air entrainment directly affects the changes in the TDG levels. Hence, the TDG level increases when the energy dissipation efficiency increases.

Moreover, the different relationships between the TDG level and these parameters are investigated. A power relationship (Eq. (6)) is adopted for TDG prediction due to its satisfactory fitting performance.

6 $$ \frac{{G_{s} - G_{f} }}{{G_{f} }} = b_{1} q^{{b_{2} }} h_{t}^{{b_{3} }} E^{{b_{4} }} + b_{5} $$

where the fitting coefficients $b_{1}$, $b_{2}$, $b_{3}$, $b_{4}$ and $b_{5}$ are 2.09, 0.033, 0.032, 0.014 and − 2.43, respectively, determined based on multiple nonlinear regression. Finally, Eq. (7) can be written as

7 $$ \frac{{G_{s} - G_{f} }}{{G_{f} }} = 2.09q^{0.033} h_{t}^{0.032} E^{0.014} - 2.43 $$

**Figure 3:**
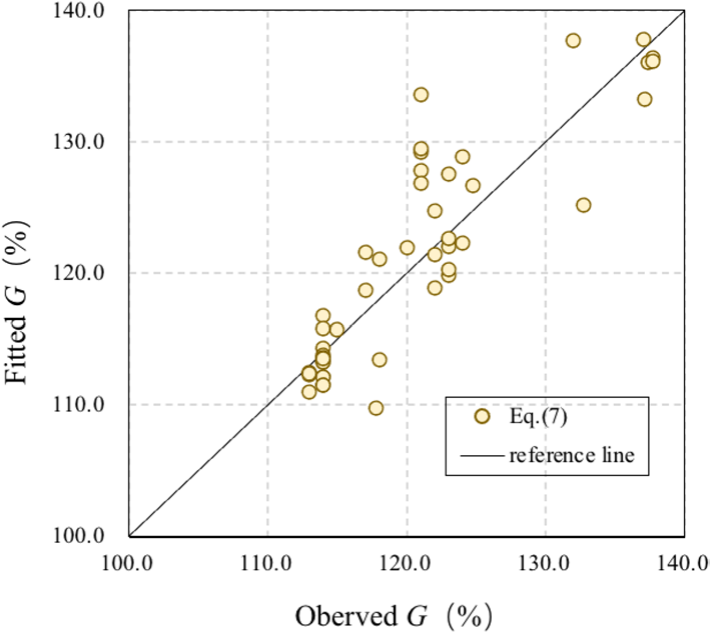
Comparison between observed and fitted G values based on Eq. (7).

Figure 3 illustrates the agreement between the fitted and observed TDG levels at different hydropower stations. The determination coefficient ($R^{2}$) is 0.68, and the average absolute error remains 3.1%.

### Analysis of the empirical equation without the energy dissipation efficiency

The coefficients in the TDG prediction equations (Eqs. (1) and (2)) proposed by USACE are calibrated with the TDG measurements in Table 1 and rewritten as Eqs. (8) and (9), respectively.

8 $$ \Delta G = 0.32h_{t} \left( {1 - e^{{0.75q_{s} }} } \right) + 21.52 $$

9 $$ \Delta G = {2}2.34h_{t}^{0.13} q_{s}^{0.14} - 50.78 $$

**Figure 4:**
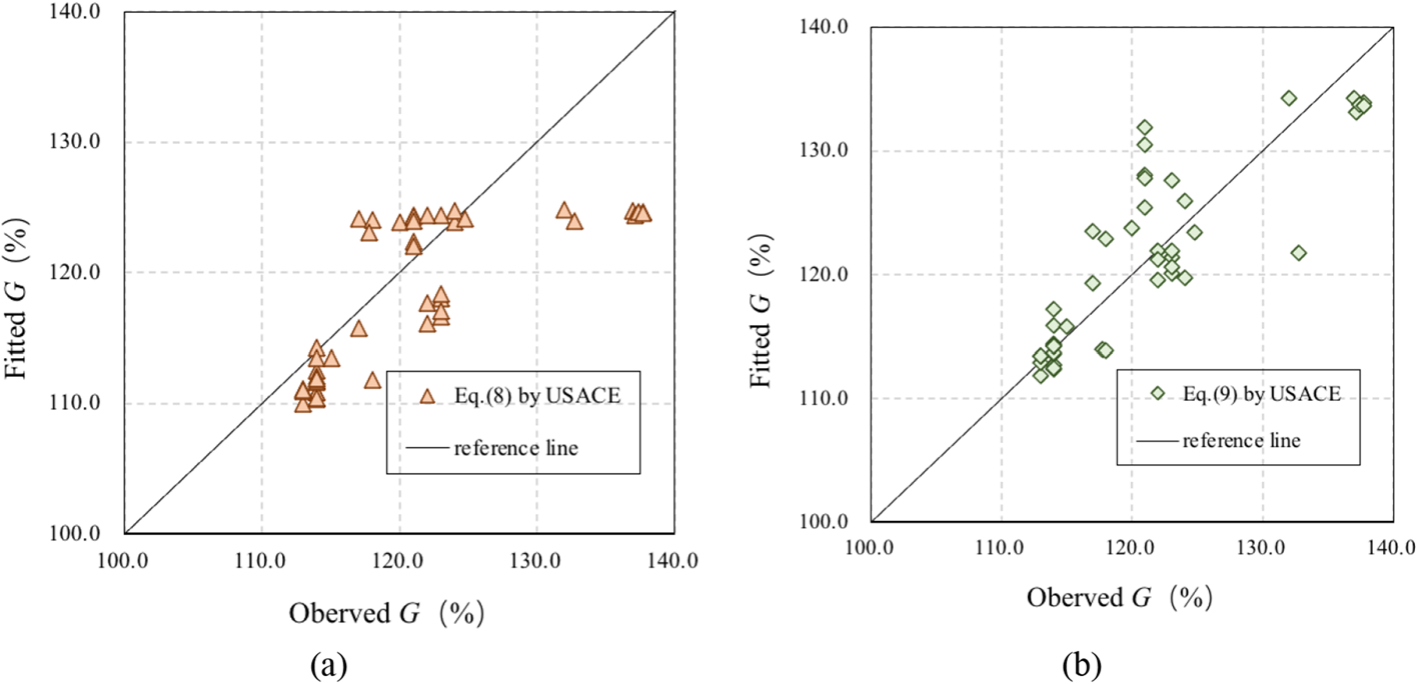
Comparison between observed and fitted G with the USACE proposed correlations: (**a**) Eq. (8); (**b**) Eq. (9).

The comparisons between the fitted and observed results are shown in Fig. 4. The determination coefficients (*R*^2^) of the observed results and fitted results based on Eqs. (8) and (9) are 0.42 and 0.68, with standard errors of 6.04% and 4.53%, respectively. Equations (8) and (9) are used to assess the TDG, as described in the next section, and the results are compared to those obtained using Eq. (7), which is established in this study.

### TDG measurements in other projects

Table 2 presents the TDG measurements in other projects. A notable gap can be observed in the TDG level between the forebay and downstream measurements. The TDG increment in the Ertan project is approximately 20%, corresponding to the maximum value among those of the considered dams. For Tongjiezi, the forebay TDG saturation level is 130% and downstream level is 148%.

**Table 2:** TDG measurements in other multiple projects.

Case	The distance between dam and observation location (km)	Release structure	Spilling rate, $Q_{s}$ (m^3^/s)	Power flow, $Q_{P}$ (m^3^/s)	Forebay water elevation (m)	Dam downstream water elevation (m)	Forebay TDG $G_{f}$(%)	Observed TDG $G$(%)
Zipingpu(a)	0.5	Discharge tunnel	170	0	865	744	107	107
Zipingpu(b)			170	0	865	744	107	115
Zipingpu(c)			170	0	865	744	107	111
Zipingpu(d)			210	0	865	743	107	112
Zipingpu(e)			210	0	865	744	107	111
Zipingpu(f)			193	0	864	744	107	112
Zipingpu(g)			210	0	864	745	107	131
Ertan(a)	2.0	Middle orifice	2054	1815	1197	1018	105	124
Ertan(b)			2044	1726	1197	1018	105	125
Ertan(c)			2026	1732	1194	1018	105	123
Manwan(a)	4.0	Surface orifice	1780	1968	990	902	105	116
Manwan(b)			1810	1930	990	902	106	114
Pubugou(a)	1.1	Discharge tunnel	643	1980	843	673	111	118
Pubugou(b)			643	2080	843	673	111	118
Tongjiezi(a)	1.6	Spillway	438	2130	470	436	130	147
Tongjiezi(b)			762	2160	470	437	127	145
Tongjiezi(c)			1079	2170	470	438	129	148
Tongjiezi(d)			629	1930	470	436	129	143
Tongjiezi(e)			800	859	472	436	129	131
Tongjiezi(f)			950	2160	472	437	130	135
Gongguoqiao(g)	0.3	Surface orifice	641	1543	1306	1247	107	110
Gongguoqiao(h)			642	1545	1306	1247	107	120
Mamaya(a)	1.0	Surface orifice	291	716	584	516	107	113
Mamaya(b)			701	801	584	517	107	119
Mamaya(c)			155	720	584	515	107	111

### Comparison of the TDG empirical correlations

**Figure 5:**
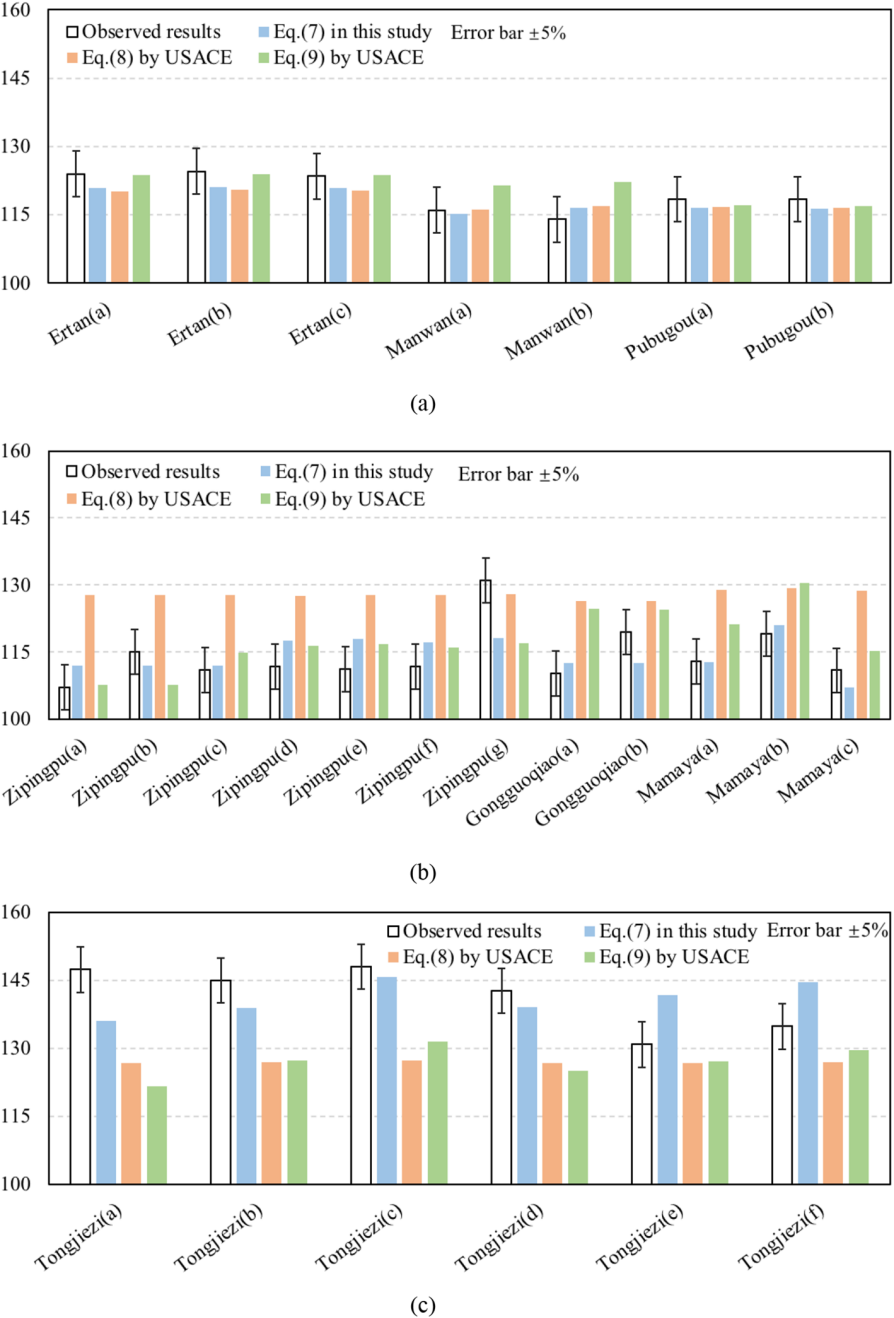
Computed TDG results by Eqs. (9), (10) and (11) compared to the observed TDG level at: (**a**) Ertan, Manwan, Pubugou projects; (**b**) Zipingpu, Gongguoqiao, Mamaya projects; (**c**) Tongjiezi project.

Figure 5 and Table 3 present the TDG estimated results based on correlations reported in the literature. Considering reasons such as instrumental and manipulation error, absolute errors of less than 5% between the predictions and observations are assumed to be acceptable.

**Table 3:** Comparison of the TDG estimation performance.

	Equation (9)	Equation (10) from USACE	Equation (11) from USACE
Determination coefficient	0.78	NaN	0.35
Absolute error	4.6% (average) 12.9% (max)	10.9% (average) 20.8% (max)	7.5% (average) 25.7% (max)
Standard error	5.7%	13.0%	10.0%
Percent of absolute error values under 5%	16/25	9/25	11/25

At the Manwan and Pubugou hydropower stations, the results fitted using Eqs. (7), (8) and (9) are not considerably different and mostly consistent with the measured TDG levels, with standard errors of 1.9%, 1.9% and 5.1%, respectively. For the Ertan project, the average absolute error pertaining to Eqs. (7), (8) and (9) is less than 5% (Fig. 5a). The TDG levels estimated using Eq. (8) are considerably higher than those observed in the Zipingpu, Gongguoqiao and Mamaya projects, with an average absolute error of 14.0%; additionally, the absolute errors between the observed TDG levels and levels predicted using Eqs. (7) and (9) are less than 5% for most projects (Fig. 5b). However, Eqs. (7), (8) and (9) cannot effectively model the scenario at Tongjiezi dam, as indicated by the large gaps between the estimated and observed TDG levels, specifically, 13.5%, 20.8% and 25.7%, respectively (Fig. 5c).

The determination coefficient for Eq. (7) (0.75) is significantly higher than those of Eqs. (8) and (9). The maximum and average values of the absolute error for Eq. (7) are 4.3 and 13.5, respectively, smaller than those for Eqs. (8) and (9). Moreover, Eq. (7) provides more reliable estimates than Eqs. (8) and (9), with 18 of 25 cases exhibiting absolute errors less than 5%.

For Eq. (7), the TDG estimate considering the energy dissipation deficiency is significantly enhanced, although the accuracy is not as high as expected. This result suggests that the energy dissipation efficiency plays a key role in the TDG prediction. In particular, energy dissipation efficiency reflects the air entrainment level. In other words, the air entrainment may be one of the main factors affecting the TDG generation process and must be incorporated in TDG prediction models in the future.

### Error analysis and uncertainty

Field observations are used to analyze the correlation between the TDG level and energy dissipation efficiency. The precision of the observed TDG level is significant in the analysis. For certain dams, it is difficult to assess the mixing level of the powerhouse and spilling flows at the observed locations. In this study, the layout of the dam and observation location are considered to determine whether the flows are completely mixed. Certain misjudgment regarding the mixing level of the powerhouse and spilling flows likely occurs, which increases the error.

Nevertheless, these correlations do not take into account the knowledge of the air–water transfer process considering the spilling conditions, and thus, the generality and accuracy of the proposed approach may be limited.

In general, this study demonstrates that the correlation between the TDG level and energy dissipation can significantly enhance the performance of the generated TDG level estimation. Moreover, the findings provide a cornerstone for further research on TDG mitigation by reasonably evaluating the TDG level. The energy dissipation efficiency reflects the air entrainment process that occurs during spilling. Future work must be aimed at analyzing the TDG generation process during dam spilling to establish a TDG mechanical prediction model that considers the air entrainment.

## Materials and methods

### Studied spillage cases

Sichuan University has measured the TDG levels at the Dagangshan and Xiluodu hydropower stations since 2015. The Xiluodu hydropower station, with the second-largest installed capacity in China, is located in the lower reaches of Jinsha River, and the Dagangshan hydropower station is situated in the middle reach of Dadu River. The TDG measurement results for these two projects and corresponding spilling characteristics are considered to analyze the relationship between the TDG and the tailwater depth, discharge per unit width and energy dissipation efficiency. Overall, 49 cases are considered in the analysis, as shown in Table 1. The TDG level is affected by the total dissolved gas pressure TGP (mmHg) and barometric pressure BP (mmHg). The TDG calculation involves the following steps: A PT4 Tracker (Point Four Systems, Coquitlam, Canada) is used to determine the TDG level. The total dissolved gas pressure TGP (mmHg) and barometric pressure BP (mmHg) are measured using the PT4 tracker, and the TDG level can be derived using Eq. (10). The measurement range of PT4 is 0–200% with an accuracy of $\pm 1{\text{\% }}$. During dam spilling, the probes of the PT4 trackers are placed in the flow in the forebay and downstream of the dam. After the readings of these parameters stabilize, TDG saturation is recorded for the given spillage case.

10 $$ TDG{\%} = \frac{TGP}{{BP}} \times 100\% $$

The TDG measurements based on the PT4 tracker from seven other dams (Zipingpu, Ertan, Manwan, Pubugou, Tongjiezi, Gongguoqiao and Mamaya) on different rivers such as Minjiang River and Lancangjiang River have been obtained by Sichuan University in recent years. These spillage cases are used to compare the estimated TDG levels when considering and not considering the energy dissipation efficiency (Table 2).

### Mixing level of the spilling flow and power flow

The mixing level of the spilling flow and powerhouse flow affects the TDG level at the observation location. We consider the Dagangshan and Xiluodu hydropower stations as examples. The layouts of the Dagangshan and Xiluodu dams and corresponding observation sites are presented in Supplementary Fig. S1, prepared using OvitalMap v8.7.0 (https://www.ovital.com/). The observation location at the Dagangshan hydropower station lies upstream of the powerhouse flow outlet (Supplementary Fig. S1a); thus, the collected TDG level ($G$) is expected to be equal to the TDG level that results from dam spilling ($G_{s}$) without power flow mixing. At Xiluodu dam, the release structure is near the powerhouse, and the distance between the observation location and dam is approximately 4.2 km (Supplementary Fig. S1b). Hence, the spilling flow and powerhouse flow are expected to be fully mixed. It is assumed that the TDG does not change during power generation; in other words, the powerhouse TDG level remains identical to the forebay TDG level^30^. In this situation, the TDG level generated by the spillage ($G_{s}$) at this dam is obtained using Eq. (11) ^11^. Moreover, the mixing level is also considered for the spillage cases listed in Table 2.

11 $$ G_{s} = \frac{{G\left( {Q_{s} + Q_{p} } \right) - G_{f} Q_{p} }}{{Q_{s} }} $$

where $G_{f}$ is the forebay TDG level (%); $Q_{s}$ and $Q_{p}$ are the spilling and powerhouse flowrates, respectively (m^3^/s).

### Energy dissipation efficiency

Energy dissipation is defined as the heat loss between the outlet section of the spilling structure (Sect. 1-1) and the section in the river downstream of the stilling basin (Sect. 2-2), as shown in Supplementary Fig. S2. In each section, the energy is related to the flow velocity, water elevation and pressure. In Sects. 1-1 and 2-2, the flow has been exposed to the atmosphere, and thus, the pressure is not considered. Finally, the energy dissipation efficiency ($E$) associated with spilling is calculated using Eq. (12) ^31^, and the variables are illustrated in Supplementary Fig. S2.

12 $$ E = 1 - \frac{{h_{t} + \frac{{v_{t}^{2} }}{{{2}g}}}}{{h_{s} + \frac{{v_{{\text{s}}}^{2} }}{{{2}g}}}} $$

where $h_{t}$ is the tailwater depth; $h_{s}$ is the release structure outlet height (m); $v_{s}$ is the velocity at the outlet of a release structure (${\text{m/s}}$);$v_{t}$ is the velocity downstream of the dam (${\text{m/s}}$).

## Supplementary Information

Supplementary Information 1.

Supplementary Information 2.
