# Supplementary material for: Energy dissipation efficiency as a new variable in the empirical correlation of total dissolved gas
Source: Sci Rep. 2021 Apr 1;11:7414. doi: 10.1038/s41598-021-86144-y (PMC8016975; doi:10.1038/s41598-021-86144-y)
Supplement: Supplementary file 1 — Supplementary Information 1. [file 41598_2021_86144_MOESM1_ESM.docx]

# ENERGY DISSIPATION EFFICIENCY as a new variable in the EMPIRICAL correlation of TOTAL DISSOLVED GAS

# Authors:

Jingying Lu^a,b^, Xiaolong Cheng^a^, Zhenhua Wang^a^, Ran Li^a^*, Jingjie Feng^a^, Kefeng Li^a^, Zhongluan Yan^c^

^a^Sichuan University State Key Laboratory of Hydraulics and Mountain River Engineering, Chengdu, Sichuan, CN.

^b^China Three Gorges Corporation, Beijing, CN.

^c^China Three Gorges Projects Development Co.,Ltd.

# *Correspondance:

Ran Li, Sichuan University State Key Laboratory of Hydraulics and Mountain River Engineering, Chengdu, Sichuan, CN.

Email: liran@scu.edu.cn


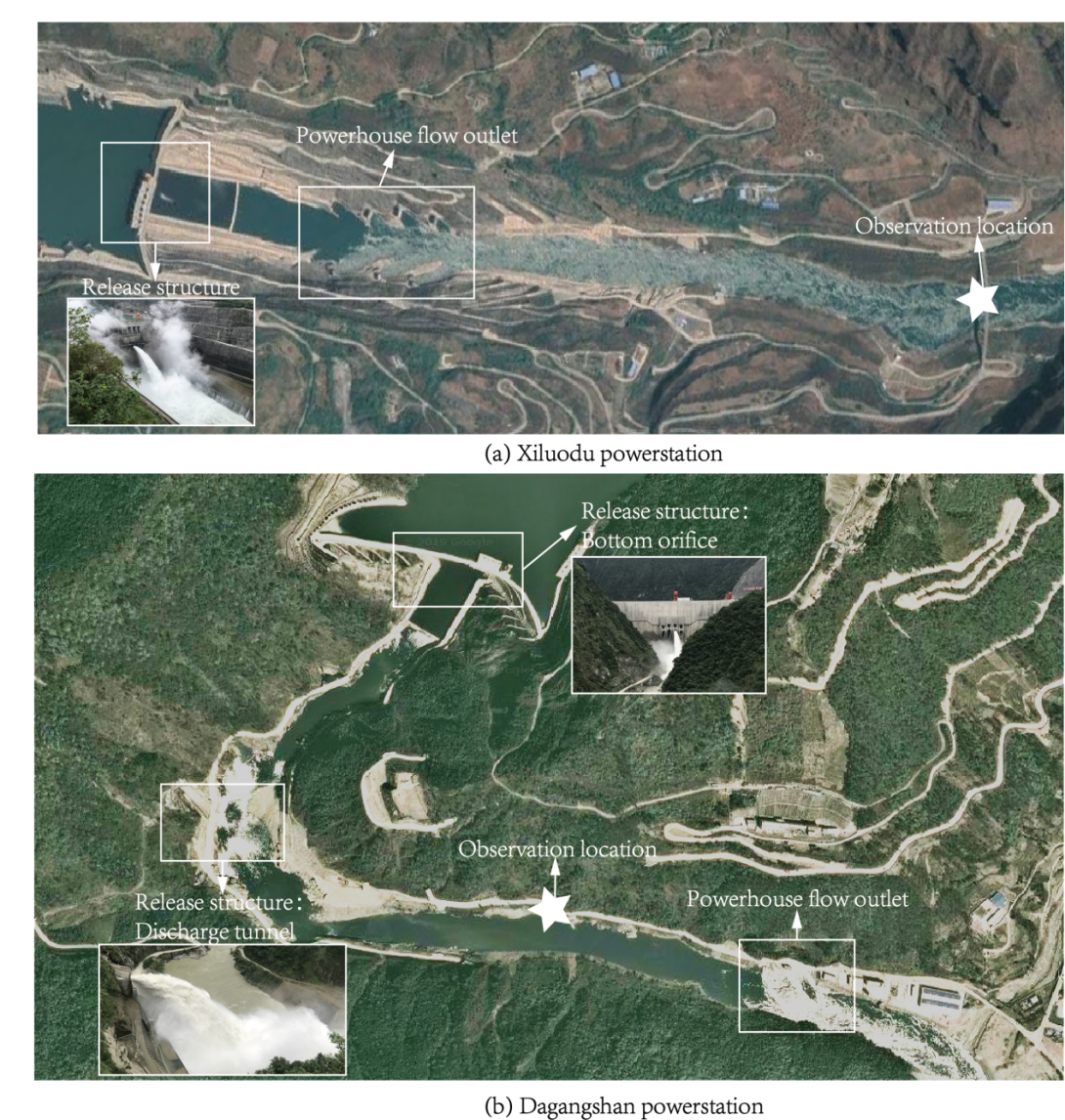


**Fig. S1** Layouts of the hydropower stations and their observation locations by OvitalMap v8.7.0 (https://www.ovital.com/):

(a) Dagangshan; (b) Xiluodu


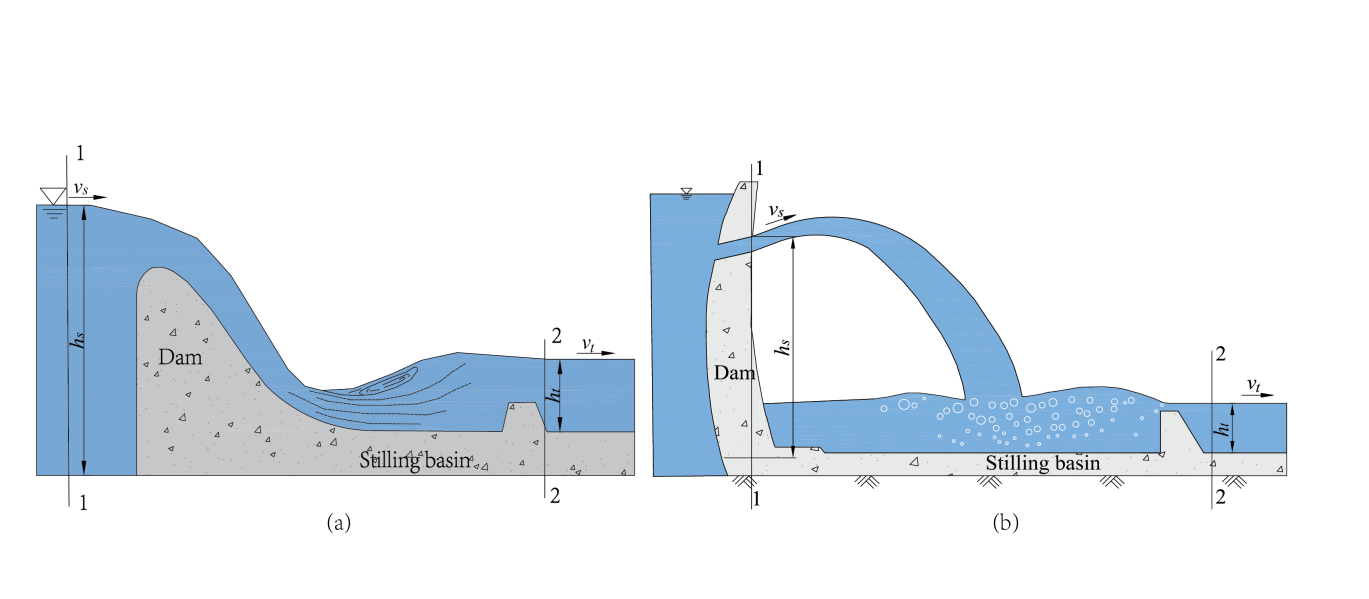


**Fig. S2** Main variables in the energy dissipation efficiency:(a) spillway; (b) plunging jet
