# Supplementary material for: Energy dissipation efficiency as a new variable in the empirical correlation of total dissolved gas
Source: Sci Rep. 2021 Apr 1;11:7414. doi: 10.1038/s41598-021-86144-y (PMC8016975; doi:10.1038/s41598-021-86144-y)
Supplement: Supplementary file 2 — Supplementary Information 2. [file 41598_2021_86144_MOESM2_ESM.docx]

Table 1 The relative parameters in TDG generation for Dagangshan and Xiluodu projects

| Case | Project | The distance between dam and observation location (km) | Release structure | Spilling rate, $Q_{s}$(m3/s) | Power flow, $Q_{P}$(m3/s) | The discharge per unit width,  *q*(m^2^/s) | Forebay water elevation,  (m) | Dam  Downstream water elevation,  (m) | The velocity of release structure outlet,  *v_s_* (m/s) | Head loss  (m) |
| --- | --- | --- | --- | --- | --- | --- | --- | --- | --- | --- |
| 1~8 | Dagangshan | 1.0 | Discharge tunnel | 713~2420 | 269~1350 | 51~173 | 1124~1129 | 954~960 | 7.1~11.8 | 164~172 |
| 9~15 |  |  | Bottom orifice | 2584~2680 | 524~1330 | 85~223 | 1121~1125 | 959~960 | 33.0~33.8 | 159~164 |
| 16~19 |  |  | Bottom orifice and discharge tunnel | 1690~2663 | 775~1230 | 104~218 | 1122~1123 | 958~959 | 14.8~33.3 | 162~163 |
| 20 | Xiluodu | 4.2 | Four bottom orifices | 5414 | 7503 | 226 | 5786 | 387 | 33.6 | 248 |
| 21 |  |  | Triple bottom orifices | 4083 | 7463 | 227 | 578 | 385 | 33.8 | 238 |
| 22~40 |  |  | Single bottom orifice | 1463~1538 | 3262~6760 | 244~256 | 591~600 | 381~384 | 36.4~37.6 | 195~217 |
| 41~49 |  |  | Double bottom orifices | 3005~3039 | 2145~6626 | 250~-253 | 595~597 | 382~385 | 37~38 | 210~211 |

| Case | Project | The distance between dam and observation location (km) | Release structure | Flow velocity downstream dam, *v_t_* (m/s) | Energy dissipation | Forebay TDG,  $G_{f}$(%) | Observed TDG,  $G$(%) | Generated TDG by spillage, $G_{s}$(%) | $\frac{G_{s}\text{-}G_{f}}{G_{f}}$ |
| --- | --- | --- | --- | --- | --- | --- | --- | --- | --- |
| 1~8 | Dagangshan | 1.0 | Discharge tunnel | 2.4~4.6 | 0.18~0.19 | 108~115 | 117~124 | 117~124 | 0.05~0.10 |
| 9~15 |  |  | Bottom orifice | 4.2~5.0 | 0.15~0.18 | 110~113 | 132~138 | 132~137 | 0.08~0.25 |
| 16~19 |  |  | Bottom orifice and discharge tunnel | 3.2~5.3 | 0.17~0.20 | 113 | 122~141 | 125~141 | 0.07~0.25 |
| 20 | Xiluodu | 4.2 | Four bottom orifices | 1.9 | 0.44 | 125 | 104 | 154 | 0.48 |
| 21 |  |  | Triple bottom orifices | 1.7 | 0.45 | 123 | 104 | 158 | 0.52 |
| 22~40 |  |  | Single bottom orifice | 0.9~1.4 | 0.47~0.49 | 113~118 | 104~107 | 158~134 | 0.28~0.61 |
| 41~49 |  |  | Double bottom orifices | 1.1~0.8 | 0.48~0.49 | 121~123 | 106~109 | 132~147 | 0.24~0.48 |

Table 2 The relative parameters in the generated cases of multiple hydropower stations

| Case | The distance between dam and observation location (km) | Release structure | Spilling rate, $Q_{s}$(m3/s) | Power flow, $Q_{P}$(m3/s) | The discharge per unit width,  *q*(m^2^/s) | Forebay water elevation,  (m) | Dam  Downstream water elevation,  (m) | | The velocity of release structure outlet,  *v_s_* (m^2^/s) | Flow velocity downstream dam, *v_t_* (m^2^/s) | Energy dissipation | Head loss (m) | Forebay TDG,  $G_{f}$(%) | Observed TDG,  $G$(%) | $\frac{G_{s}\text{-}G_{f}}{G_{f}}$ |
| --- | --- | --- | --- | --- | --- | --- | --- | --- | --- | --- | --- | --- | --- | --- | --- |
| Zipingpu(a) | 0.5 | Discharge tunnel | 170 | 0 | 20 | 865 | | 744 | 16.2 | 0.1 | 0.14 | 121 | 107 | 107 | 0.00 |
| Zipingpu(b) |  | Discharge tunnel | 170 | 0 | 20 | 865 | | 744 | 16.2 | 0.1 | 0.14 | 121 | 107 | 115 | 0.07 |
| Zipingpu(c) |  | Discharge tunnel | 170 | 0 | 20 | 865 | | 744 | 16.2 | 0.1 | 0.14 | 121 | 107 | 111 | 0.04 |
| Zipingpu(d) |  | Discharge tunnel | 210 | 0 | 39 | 865 | | 743 | 16.6 | 0.1 | 0.14 | 122 | 107 | 112 | 0.04 |
| Zipingpu(e) |  | Discharge tunnel | 210 | 0 | 39 | 865 | | 744 | 28.5 | 0.1 | 0.14 | 120 | 107 | 111 | 0.04 |
| Zipingpu(f) |  | Discharge tunnel | 193 | 0 | 36 | 864 | | 744 | 26.1 | 0.1 | 0.14 | 120 | 107 | 112 | 0.04 |
| Zipingpu(g) |  | Discharge tunnel | 210 | 0 | 39 | 864 | | 745 | 28.5 | 0.1 | 0.14 | 119 | 107 | 131 | 0.22 |
| Ertan(a) | 2.0 | Middle orifice | 2054 | 1815 | 171 | 1197 | | 1018 | 34.2 | 0.4 | 0.15 | 179 | 105 | 124 | 0.35 |
| Ertan(b) |  | Middle orifice | 2044 | 1726 | 170 | 1197 | | 1018 | 34.1 | 0.4 | 0.15 | 179 | 105 | 125 | 0.35 |
| Ertan(c) |  | Middle orifice | 2026 | 1732 | 169 | 1194 | | 1018 | 33.8 | 0.4 | 0.09 | 177 | 105 | 123 | 0.33 |
| Manwan(a) | 4.0 | Surface orifice | 1780 | 1968 | 137 | 990 | | 902 | 6.8 | 0.7 | 0.07 | 88 | 105 | 116 | 0.22 |
| Manwan(b) |  | Surface orifice | 1810 | 1930 | 139 | 990 | | 902 | 7.0 | 0.7 | 0.20 | 69 | 106 | 114 | 0.16 |
| Pubugou(a) | 1.1 | Discharge tunnel | 643 | 1980 | 54 | 843 | | 673 | 3.3 | 0.2 | 0.20 | 167 | 111 | 118 | 0.27 |
| Pubugou(b) |  | Discharge tunnel | 643 | 2080 | 54 | 843 | | 673 | 3.3 | 0.2 | 0.15 | 167 | 111 | 118 | 0.28 |
| Tongjiezi(a) | 1.6 | Spillway | 438 | 2130 | 31 | 470 | | 436 | 7.8 | 0.2 | 0.07 | 34 | 130 | 147 | 0.13 |
| Tongjiezi(b) |  | Spillway | 762 | 2160 | 54 | 470 | | 437 | 6.8 | 0.3 | 0.07 | 33 | 127 | 145 | 0.14 |
| Tongjiezi(c) |  | Spillway | 1079 | 2170 | 77 | 470 | | 438 | 3.9 | 0.4 | 0.07 | 33 | 129 | 148 | 0.15 |
| Tongjiezi(d) |  | Spillway | 629 | 1930 | 45 | 470 | | 436 | 11.2 | 0.2 | 0.07 | 34 | 129 | 143 | 0.10 |
| Tongjiezi(e) |  | Spillway | 800 | 859 | 57 | 472 | | 436 | 11.4 | 0.3 | 0.08 | 36 | 129 | 131 | 0.01 |
| Tongjiezi(f) |  | Spillway | 950 | 2160 | 68 | 472 | | 437 | 11.3 | 0.3 | 0.07 | 35 | 130 | 135 | 0.04 |
| Gongguoqiao(g) | 0.3 | Surface orifice | 641 | 1543 | 43 | 1306 | | 1247 | 10.8 | 0.3 | 0.04 | 59 | 107 | 110 | 0.03 |
| Gongguoqiao(h) |  | Surface orifice | 642 | 1545 | 43 | 1306 | | 1247 | 10.8 | 0.3 | 0.04 | 59 | 107 | 120 | 0.12 |
| Mamaya(a) | 1.0 | Surface orifice | 291 | 716 | 20 | 584 | | 516 | 13.4 | 0.2 | 0.12 | 69 | 107 | 113 | 0.06 |
| Mamaya(b) |  | Surface orifice | 701 | 801 | 48 | 584 | | 517 | 12.4 | 0.5 | 0.12 | 67 | 107 | 119 | 0.11 |
| Mamaya(c) |  | Surface orifice | 155 | 720 | 11 | 584 | | 515 | 13.4 | 0.1 | 0.12 | 69 | 107 | 111 | 0.04 |
